# Knowledge and perceptions towards malaria prevention among vulnerable groups in the Buea Health District, Cameroon

**DOI:** 10.1186/1471-2458-14-883

**Published:** 2014-08-27

**Authors:** Helen K Kimbi, Sarah B Nkesa, Judith L Ndamukong-Nyanga, Irene UN Sumbele, Julius Atashili, Mary BS Atanga

**Affiliations:** Department of Zoology and Animal Physiology, University of Buea, Buea, Cameroon; Department of Public Health and Hygiene, University of Buea, Buea, Cameroon; Department of Nursing, University of Bamenda, Bamenda, Cameroon

**Keywords:** Malaria, Knowledge, Bed nets, Pregnant women, Children, Cameroon

## Abstract

**Background:**

Malaria is a public health problem especially in vulnerable groups such as pregnant women and children under five years in Cameroon including the Buea Health District (BHD). Misconceptions concerning it exist. This study assessed the level of knowledge and perceptions towards malaria control among pregnant women and mothers/caretakers of under-fives in the BHD.

**Methods:**

A community-based cross-sectional study was conducted in the BHD in August, 2011 in five health areas. A questionnaire was used to collect data on demographic variables, knowledge and perceptions towards malaria control from 443 respondents aged 15–73 years.

**Results:**

Of the 443 respondents interviewed, 99% had heard about malaria. Awareness of malaria was similar in rural (98.04%) and urban (98.97%) areas. The health facility was the most popular source of information (74%). The radio, television, tracts/posters and the community relay agents (CRAs) all informed significantly higher proportions of respondents in the urban than rural communities (P <0.05). Overall, 92% of respondents had the right perception of malaria and 88% knew at least one correct sign/symptom of malaria. The most recognised sign of malaria was fever. When all aspects of malaria were considered, majority (88%) of respondents had good levels of knowledge on malaria. The level of good knowledge in respondents with **≥** secondary school education (91%) was significantly higher (P = 0.01) than in those with ≤ primary school level (83%). Overall, 99% had heard about insecticide treated nets (ITNs); 99% perceived ITNs as a good means to prevent malaria; most respondents **(**57%) used ITNs mainly for protection against mosquito bites while 48% used them for protection against malaria.

**Conclusion:**

Respondents with no formal education had a poor level of knowledge on malaria. Hence, new strategies for sensitization messages involving their active participation need to be developed.

## Background

Malaria is a serious public health problem in sub-Saharan Africa. It affects the health and wealth of nations and individuals alike [1]. The vast majority of estimated cases (80%) and deaths (91%) occur in sub-Saharan Africa and the vast majority of deaths (86%) occur in children <5 years of age [2]. Malaria remains inextricably linked with poverty [2].The human and economic costs associated with the declining quality of life, consultations, treatments, hospitalizations and other events related to malaria are enormous and often lead to low productivity and loss of income [3]. Children aged less than five years and pregnant women are the people most vulnerable to dying of malaria or suffering serious consequences of the disease, especially in regions where transmission is intense [4]. Children are most vulnerable because they have not acquired immunity to the disease [5], while maternal susceptibility to malaria infection during pregnancy may be related to the physiological immunosuppression that occurs during gestation [6].

Malaria infection during pregnancy can have adverse effects on both mother and foetus, including maternal anaemia, foetal loss, premature delivery, intrauterine growth retardation, and delivery of low birth-weight infants which is a risk factor for death [7]. In children under five, the adverse effects include: convulsions, anaemia, coma and death [8]. Preventing malaria is of prime importance in reducing the rates of morbidity and mortality in the country. Malaria is responsible for 31% of consultations, 44% of hospitalizations and 18% of deaths occurring in health facilities in the country. In children less than 5 years, 41% of deaths are due to malaria [9]. If malaria is appropriately prevented, the individual, family and the state will save lots of resources that will improve the standards of living of the general population. The practice of malaria preventive measures has been related to the level of knowledge and belief of people. The understanding of the possible causes, modes of transmission and decision about adoption of preventive and control measures vary from community to community and among individual households [3, 10, 11]. The current emphasis on malaria control is centred on community-based strategies [12, 13]. In order to prepare for a successful malaria control program it is necessary to evaluate the level of awareness, attitudes and practices of people living in an at risk area. This will help to find ways to improve collaboration with the public health sector [14] and also involve the full participation of the community in surveillance and control activities such as the use of ITNs/ long lasting insecticide treated bed nets [15]. Capacity needs to be built in residents of such areas to empower them with adequate knowledge aimed at behaviour change and selection of appropriate control measures against malaria. This could be done through mother and child health programs. There have been several reports about knowledge, attitude, and practice relating to malaria and its control from different parts of Africa [3, 16–18]. Nkuo-Akenji *et al.* [19] also reported that an adequate knowledge of mothers of under-fives about malaria had a great correlation with reduced morbidity and mortality among children less than five in Bolifamba, Cameroon. However, a lot of misconceptions concerning malaria still exist in such areas.

The control of malaria by the National Malaria Control Program (NMCP) in Cameroon involves among others vector control such as; combining treated bed net use and indoor residual spraying which are locally improved with larvae control and environmental management, free distribution of intermittent preventive treatment (sulphadoxine-pyrimethamine) to pregnant women and free treatment of children aged under five with artemisinin based combination therapy [20]. However, report indicates control of malaria in pregnant women in the South West Region could hardly reach 20% coverage of the population. As such, practices for the control of malaria have been unsatisfactory despite the serious adverse effects of the disease in the BHD in the South West Region. Reported adverse effects of the disease such as anaemia may range from 14% in adults [21] to 80 % in children [22] thereby necessitating an evaluation of the consciousness of the disease. This study was therefore aimed at collecting baseline information on the level of knowledge towards the mode of transmission and prevention of malaria among pregnant women and mothers/caretakers of under-fives in the BHD. Additionally; we intended to assess the level of awareness and perception of ITNs as a preventive measure against malaria.

## Methods

### Study area

A community-based cross-sectional study was conducted in the BHD in August, 2011. Buea is the capital of the South West Region of Cameroon and is located on the eastern slopes of Mount Cameroon. BHD is circumscribed within the administrative boundaries of Buea Sub-Division and has a population of 133092 inhabitants living in seven health areas. The BHD consists of seven health areas made up of sixty-seven communities. Some are rural while others are urban. Five of the seven health areas were randomly chosen for the study namely: Bokwango, Bova, Buea Road, Muea and Tole.

The inhabitants of Buea comprise mainly of the Bakweri indigenes. However, there are a significant number of other tribes and ethnic groups such as the Bantu, Semi-Bantu and Foulbe [23] from all over the country as well as foreigners. People living in Buea perform various activities, but the majority carry out farming, business and studies. Tea cultivation constitutes a significant local industry in the Tole health area. The climate of Buea generally, is of the equatorial type with temperatures ranging from 18°C-29°C annually with average humidity of 80 %.There are two main seasons; the rainy season which starts from mid-March to November and the dry season which lasts for the rest of the year. Human malaria is *meso*-endemic during the dry season but becomes hyper-endemic in the rainy season, with incidence peaking in July-October. The prevalence of malaria parasitaemia in the Mount Cameroon region varies from 60.6%, in lowland altitude to7.7% in the highlands [24].

### Study population

The study participants were pregnant women and mothers/care-givers of under-fives in selected households in five of the seven health areas in BHD. A total of 443 participants were recruited.

### Study design

The study was a descriptive cross-sectional design. A pre-tested structured questionnaire and an observational checklist were used as data collection tools. A team of six interviewers was trained by the researchers for two days before the start of data collection on the tools to be used, purpose of the study and how to approach respondents and obtain consent. Data was collected by face to face interviews of respondents.

A household was defined as: a married man, his wife (wives) and all of his dependents who currently live with him (including biological children, adopted children, domestic workers, other family members for whom he is responsible); an unmarried (widowed, divorced, never married) woman who is recognized as the head of household and all of her dependents who currently live with her; or two or more unmarried adult persons who sleep in the same dwelling unit and who share meals (e.g. university students who share an apartment). The first thing in each eligible household was to identify the mother of an under-five or a pregnant woman. In cases where the mother could not be met on two different visits, the father or eldest mature household member (at least 15 years) was recruited as the respondent. The purpose of the study was very carefully explained to the respondent and his/her verbal informed consent was obtained before the questionnaire was administered.

The first part of the questionnaire concentrated on demographic variables which included health area, community of residence, type of settlement (rural or urban), age, sex, level of education, marital status, religion and profession, household composition and house construction materials. The second part was designed to capture knowledge, attitudes and practices towards malaria, recognition of symptoms, malaria transmission and prevention.

#### Sampling method

A multistage sampling method was used for this study. Five health areas were randomly selected; Bokwango, Buea Road, Bova, Tole and Muea. From each of the five areas chosen, six communities were randomly chosen by picking from the listing of all the neighbourhoods that make up each health area. In each randomly selected community, households with at least one pregnant woman/or a child under five were visited. The number of households to be visited for each health area and for each community was determined by considering the proportion of the population of the health area/community to the total population under study. The following procedure was used to select each household: Firstly, members of the research team stood at the centre of each street in the community and a direction was randomly chosen by spinning a bottle and the direction of the head of the bottle was chosen. Depending on the number of households found in the direction of the bottle head, each household with a pregnant woman and or child under five was visited. At the end of the direction, if the target was not attained, the team members returned to the starting point and took the opposite direction and followed the same technique until the target for the community was reached.

#### Ethical considerations

An ethical clearance for this study was obtained from the University of Buea Faculty of Health Sciences institutional review board. In addition, an administrative authorization was obtained from the Regional Delegation of Public Health, South West Region. The traditional chiefs of the various communities were also contacted and their authorizations obtained before entry into the communities. Potential respondents gave verbal consent after they had been given an explicit explanation of the study and an opportunity to ask and respond to any questions. The respondents were free to decline answering any question or totally withdraw if they so wished at any time.

### Statistical analysis

Data collected were entered into Epi info, version 3.5.1 (WHO/CDC, Atlanta, USA) and backed-up in MS Excel 4.0 (Microsoft Inc, Seattle, USA). It was then transferred to STATA 9 (STATA Corps, Texas, USA) for cleaning and analysis. Uni-variate analysis was done using simple frequency calculations and means/medians. Bi-variate analysis and comparisons of proportions between groups were done with the chi square (χ^2^) and Fisher’s exact tests (F) at 95% confidence interval (CI). Association of demographic factors of participants with correct knowledge of malaria was assessed using odds ratio (OR). The statistical level of significance was set at P <0.05.

## Results

### Demographic characteristics of participants

Overall, 443 people aged 15 to 73 were included in the study; majority (290, 65%) of whom were urban dwellers; while the rest were rural dwellers (153, 35%). The single largest group of interviewees were indigenous Bakweris (144, 33%) while others (299, 67%), were from many tribes in the country. A total of 380 (85.8%) participants were pregnant women/mothers of under-fives, while 46 (10.4%) were fathers and 17 (3.8%) were elder brothers/sisters of the under-fives. In all, 394 (88.9%) were females and with regard to the level of education, 55 (12.4%) of the respondents had attended university, 65 (14.7%) high school, 142 (32.1%) secondary, 159 (35.9%) primary and 20 (4.5%) had no formal education at all. Overall, 289 respondents (65.2%) were married while 137 (30.9%) were single. The respondents were mostly christians (438, 99%). Respondents were involved in many activities including largely, farming, hair dressing, tailoring, house-wives, health personnel, students, teaching, and small businesses.

### Composition of households

Of the 443 households visited, the total number of people in each ranged from 1–17 with a median of 5 persons per household. Children under five years were reported to be living in 391 households ranging from 1–6 children with a median of 2 children per household. One hundred and forty-eight (148) households reported the presence of at least one pregnant woman ranging from 1–5 pregnant women with a median of 1 pregnant woman per household.

### Awareness and sources of information on malaria

Of the 443 respondents, 437 (98.6%) had heard about malaria before while 6 (1.4%) said they had not. Awareness of malaria was similar in the rural (98.04%) and urban (98.97%) areas (F = 0.42).

**Figure 1:**
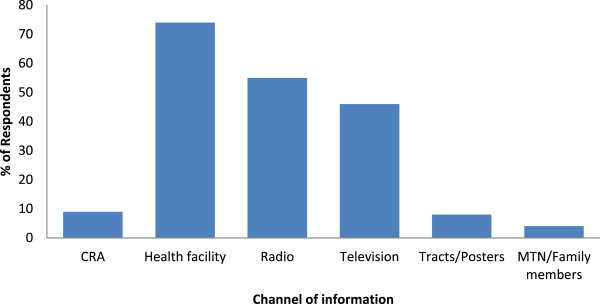
**Channels of information on malaria as stated by the study participants.**

Following multiple entries, the health facility was the most popular source of information (74% of respondents), followed in decreasing order by the radio (55%), television (46%), tracts/posters (8%) and other sources such as relatives and mobile telephone network (MTN - 4%) as shown in Figure 1.

The health facility was the only channel of information that was more popular in the rural (78%) than in the urban (72%) settlement, but the difference was not statistically significant (χ^2^ = 2.34, P = 0.13) as shown in Table 1. The radio, television, tracts/posters and the CRAs all informed significantly higher proportions of respondents in the urban than in the rural communities (Table 1).

**Table 1:** **Association of rural and urban communities with sources of information about malaria**

Source of information about malaria	% respondents		Statistics	
	Rural	Urban	χ^2^	P-value
Radio	44	61	11.55	0.001
Television	38	50	5.90	0.02
Health facility	78	72	2.34	0.13
Tracts/posters	2	11	1.33	0.001
CRA	3	12	9.44	0.002
MTN/Family members		4	0.77	0.78

### Participants’ impressions about malaria

Following the varied views of malaria by the respondents, their perceptions were grouped into three categories: right perception of malaria, wrong perception and “don’t know”/no responses. Correct views of malaria ranged from simply, malaria is “a very bad disease”, “a dangerous disease”, “a killer disease”, to “a disease associated with the bites of female *Anopheles* mosquitoes”. Wrong perceptions included: malaria is: “an air-bone disease”, “a virus in the blood”, “a disease of palm wine”, “dirty environment” among others. Overall, 407 (91.9%) of respondents had the right perception of malaria while 13 (2.9%) had wrong perceptions and 23 (5.2%) did not know what malaria is.

### Knowledge of signs and symptoms of malaria

A total of 389 (87.8%) respondents knew at least one correct sign/symptom of malaria. Thirty-six (8.1%) respondents gave wrong responses while 18 (4.1%) said they didn’t know any sign or gave no response. The most popular sign of malaria was hot body/high temperature followed by headache, nausea/vomiting, body weakness and body/joint pains. Wrong signs/symptoms included yellow urine, swollen eyes, catarrh and cough.

### Participant’s knowledge of the effects of malaria on children under five and pregnant women

**Figure 2:**
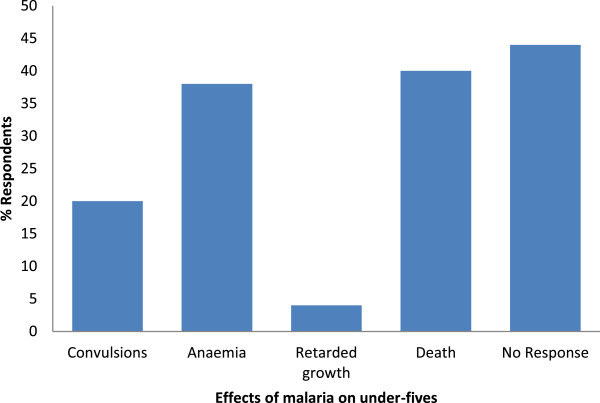
**Knowledge on the effects of malaria in children under five.**

**Figure 3:**
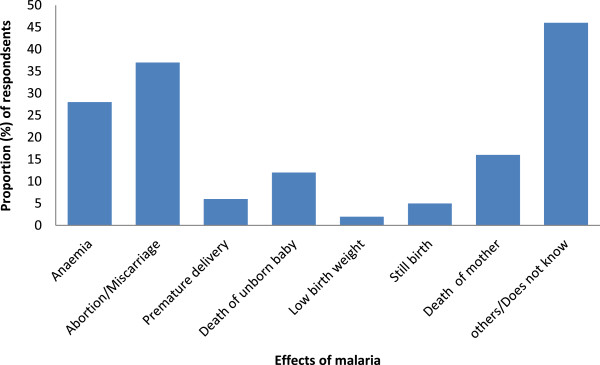
**Knowledge on the effects of malaria in pregnant women.**

The respondents had a good knowledge of the effects of malaria in children less than five and pregnant women. Of the 443 respondents, 329 (74.3%) and 294 (66.4%) knew at least one effect of malaria in children under five and pregnant women respectively while 114 (25.7%) and 149 (33.6%) gave either wrong effects or no responses for children under five and pregnant women respectively. The most popularly cited effects of malaria in children less than five were anaemia, death and convulsion while those in pregnant women included abortion, anaemia, foetal and maternal deaths as shown in Figures 2 and 3 respectively. The other cited effects included: nerve problems, persistent fever, under-nutrition and collapse.

### Participants’ perception and mode of transmission of malaria

When asked whether respondents agreed or disagreed with the fact that malaria is a dangerous disease, 240 (54.2%) strongly agreed, 172 (38.9%) agreed, 17 (3.8%) were neutral, 9 (2%) disagreed while 5 (1.1%) strongly disagreed. The 412 (93%) of the respondents who at least agreed to the fact that malaria is a dangerous disease gave reasons to support their perception such as malaria disturbs daily activities, malaria is expensive and difficult to treat, causes anaemia, abortion and many deaths.

A total of 382 (86.2%) of respondents stated the correct mode of transmission (mosquito bites), 45 (10.2%) gave wrong responses such as drinking dirty water, eating dirty food, always by the fire side, inadequate body hygiene and bites of other insects. Sixteen (3.6%) of the respondents gave no responses.

### Methods of prevention of malaria

Methods of prevention of malaria as stated by respondents reflected the mode of transmission stated. Of the 443 respondents, 413 (93.2%) stated correct measures of preventing malaria, 13 (2.9%) gave wrong measures and 17 (3.8%) gave no answer. Some of the correct measures stated included: use of mosquito bed nets and insecticidal sprays, keep the environment clean, drain stagnant water and clear bushes around homes while, the wrong preventive measures included: avoiding cold, dirty drink/food, going for frequent medical checks, wearing clean clothes, avoiding drying clothes on grass and taking nivaquine every three months.

### Association of demographic factors of participants with correct knowledge of malaria

Overall, the categories of correct and wrong/no responses for all the six aspects of knowledge of malaria discussed above (meaning of malaria, signs and symptoms of malaria, effects of malaria in under-fives and pregnant women, mode of transmission and measures of prevention) were put together to assess the level of knowledge of malaria among the participants. A participant who had correct responses for at least four of the components was considered to have a good level of knowledge while those with correct responses to three and two or less responses were considered to have average and poor levels of knowledge respectively. The majority, 390 (88%) of respondents had good, while 30 (6.8%) and 23 (5.2%) had average and poor levels of knowledge on malaria respectively.

At least 79% of respondents in all the different categories had a good knowledge of malaria as shown in Table 2**.** The proportion decreased with age from 93% in the ≥ 51 years to 79% in the 15–20 years old participants, but the difference was not signifcant (F = 0.09). The level of good knowledge in secondary/high school/university level respondents (91%) was significantly higher (χ^2^ = 6.60; P = 0.01) than in none/primary level respondents (83%). Respondents with secondary/high school/university level of education were 2.1 times (OR = 2.12, 95% CI: 1.18 – 3.78) more likely to have a good level of knowledge on malaria than their none/primary level counterparts.The level of knowledge on malaria in urban (90%) and rural (85%) respondents were comparable. Buea Road (93%) had the highest proportion of respondents and Muea (83%) had the least level of good knowledge on malaria, but the difference was not statistically significant.

**Table 2:** **Association of demographic factors of participants with correct knowledge of malaria**

Demographic variable	Category	Statistics			
		N	n (%)	Statistical test	P-value
Age (years)	13-20	58	46 (79)	Fisher’s exact	0.087
	21-50	367	328 (89)		
	≥51	15	14 (93)		
Relationship	Mother	380	336 (88)	χ^2^ = 0.38	0.540
	Father/sister/brother	63	54 (86)		
Level of education	None/primary	178	148 (83)	χ^2^ = 6.60	0.010
	Secondary/high school/university	263	240 (91)		
Profession	Non-income earners	206	177 (86)	Fisher’s exact	0.585
	Income earners	210	210 (88)		
	Health personnel	13	12 (92)		
Settlement	Rural	153	130 (85)	χ^2^ = 2.90	0.148
	Urban	290	260 (90)		
Health area	Bokwango	68	60 88)	χ ^2^ = 8.43	0.077
	Bova	44	37 (84)		
	Buea Road	160	14 (93)		
	Muea	126	105 (83)		
	Tole	45	39 (87)		
Sex	Male	49	43 (88)	χ^2^ = 0.003	0.954
	Female	393	346 (88)		

N = number interviewed; n = number knowledgeable.

### Awareness of ITNs as a preventive measure against malaria

**Figure 4:**
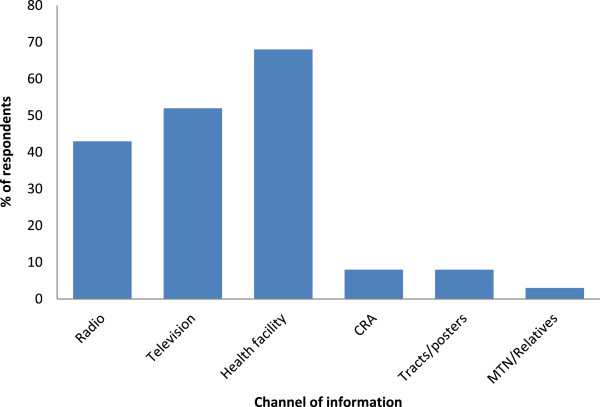
**Channels of information on ITNs.**

Out of the 443 respondents, 208 (47.0%) possessed at least one mosquito bed net (total = 275) with a median of 1.33 nets. Of the 275 nets found in households, 89 (32%) were potent ITNs and others had never been retreated/treated**.** The level of awareness of ITNs as a preventive measure against malaria was quite high among respondents. Of the 437 respondents, 432 (98.9%) had heard about ITNs before. Similarly as for malaria, the sources of information about ITNs were the same as seen in Figure 4. The most accessible channel was the health facility (302, 69.1%), followed by the television (231, 52.9%) and the radio (190, 43.5%).

### Perception of ITNs as a preventive measure against malaria

ITNs were generally perceived as a good means to prevent malaria (99.0%, 433). Only 0.9% (4) of respondents perceived ITNs as a bad preventive measure against malaria.

### Participants’ perceived benefits of ITNs

**Figure 5:**
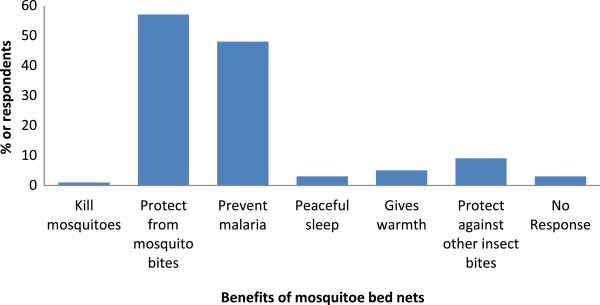
**Level of knowledge of benefits of mosquito bed nets to users.**

The stated benefits of using ITNs are shown in Figure 5. Most respondents (257, 58.8%) used ITNs mainly to protect themselves from mosquito bites while 213 (48.7%) used them with the belief that they would be protected from malaria. It can be observed that a good proportion of respondents did not know the various benefits of ITNs. It is important to note that the insecticidal property which is the main function of ITNs had the lowest percentage of respondents stating it.

## Discussion

This study assessed the level of knowledge, and perceptions towards malaria prevention in the BHD in pregnant women and mothers/care takers of under-fives. The study revealed that majority (99%) of respondents had heard about malaria before. This is not surprising as the BHD is an endemic area for malaria and it is expected that the inhabitants of such an area who frequently suffer from bouts of malaria should be knowledgeable about the disease. This agrees with the report of Mazigo *et al*. [25] in Tanzania and Hanafi-Bojd *et al*. [14] in Iran. Despite the high level of awareness, asymptomatic malaria cases still remain relatively high in the BHD [26]. Mboera *et al*. [27] also remarked that despite a high level of malaria awareness in Tanzania, malaria morbidity remained high. They attributed this to the injudicious use of antimalarial drugs, delayed health seeking behaviour, and reliance on clinical judgment without laboratory confirmation in most of the peripheral health facilities. However, with the expansion in coverage and distribution of ITNs to every household by the national malaria control program, the presence of asymptomatic malaria cases and malaria morbidities are expected to decline as reported in other studies [28, 29].

The similarity in malaria awareness in rural (98%) and urban (99%) areas attests to the fact that malaria constitutes a major health problem in both settings. Erhun *et al*. [3] and Mazigo *et al*. [25] reported similar findings in Nigeria and Tanzania respectively. However, Rakhshani *et al.* [30] reported that awareness among rural housewives was higher than in those of urban areas.

Malaria was perceived by majority (93%) of respondents as dangerous while 7% were somewhat indifferent. This latter category may be at risk of suffering from severe malaria as they may not bother to prevent or seek for treatment on time. It is worrisome to know that despite the dangerous nature and consequences of malaria some members of the study area were not well informed of the multi-dimensional challenges of the disease. This shows that the knowledge of the community is far from perfect and some misconceptions still exists in the population.

The health facility was the most popular channel of information on malaria for both rural and urban settlements. This implies that health workers at different levels of health care delivery systems stand good chances of disseminating relevant information about malaria and mosquito vectors within the community. This is in line with the report of Hanafi-Bojd *et al*. [14] in Iran. This awareness could be increased by focusing on media announcements, organizing short seminars at meetings in schools and in offices [17, 31].

The awareness of the correct mode of transmission of malaria by respondents was good (86%). However, it contrasts sharply with a similar study carried out in Ndu - North West Region of Cameroon 9 years earlier where Nsagha *et al.* [32], recorded a 27.9% correct knowledge of malaria transmission. This could be attributed to the fact that Nsagha *et al.* [32] interviewed the general population while our study focused on pregnant women and mothers/care-takers of under-fives who may be more exposed to malaria attacks and so visit health facilities more often where they receive messages on malaria. In addition, Ndu is more of a rural community than Buea which is largely urban and has better access to many different channels of information such as radio, television and CRAs. As in the study in Ndu, participants in the BHD also stated unclean water/food and palm wine as causes of malaria. Misconceptions have been reported in other studies in Nigeria (3,17,33] and Ethiopia [10]. Such misconceptions may arise from the way health education about malaria prevention and control is provided to the community. For example, Erhun *et al*. [33] reported that the key message ‘keeping environmental sanitation’ is commonly used in malaria prevention and control interventions and the meaning of environmental sanitation may be different for different people and may be interpreted differently beyond mere action for prevention of malaria.

Respondents were equally well knowledgeable on the signs/symptoms and measures of prevention of malaria. Hot body/high temperature was the most stated sign and symptom. Most respondents (92.7%) in a study in Ethiopia [34] and Tanzania [25] were able to name at least one symptom of malaria. Similarly, Erhun *et al*. [3] also reported that most respondents in his study in Nigeria were familiar with the signs and symptoms associated with malaria as is expected for any population in a malaria endemic area.

The effects of malaria in under-fives and pregnant women were well known to respondents. The prominent effects on pregnant women were anaemia, abortion, intrauterine death and maternal death. Meanwhile, death, anaemia and convulsion were stated as the effects in children below five. This implies that mothers/guardians of under-fives and pregnant women are quite aware of the dangers that malaria poses and it is expected that they would embrace any measure that will enable them preserve their pregnancies, health, lives and those of their infants.

The knowledge of malaria was strongly associated with the level of formal education. This can be explained by the fact that those who have attained at least secondary level of education might have been taught lessons on malaria in school, and are also more likely to read and comprehend malaria messages on tracts, radio or television. Education remains a powerful tool that empowers people to enable them make decisions for themselves and influence their families. Education clearly influences knowledge about modes of malaria transmission. Educated communities also have multiple sources of information when compared with their uneducated counterparts [3, 17]. Hanafi-Bojd *et al*. [14] also found a significant correlation between the education level of respondents and their interest in participating in malaria control programs as volunteers in Iran. They also were of the opinion that an increasing trend in literacy is a protective factor for malaria morbidity. Special strategies such as community educators are therefore required to reach out to the uneducated members of the study area with information, education and communication messages on malaria control measures such as ITN use, care and maintenance of ITN life span for protection against malaria [35]. Educational messages targeted, especially towards uneducated mothers/pregnant women and stressing the potential severe consequences of fever illness in children could lead to a change in health seeking behaviour and control strategies. This could be achieved by provision of comprehensive behaviour change communication, through the local media in a language accessible and appropriate to economically vulnerable populations [3].

There was a wide acceptance of mosquito bites as the cause of malaria. This relatively high level of knowledge was probably due to frequent bites from mosquitoes in the area which is endemic for malaria. The level of awareness of ITNs as a preventive measure against malaria was quite high (99%) among respondents. Top on the list of preventive measures stated was prophylaxis, ITNs, clearing of bushes and environmental sanitation. However, only 23 (5.2%) respondents mentioned the drainage of standing water, which is the one of the most important measures to control mosquito breeding. Respondents viewed ITNs mainly as a means of protection from mosquito bites (57%) and secondly for preventing malaria (48%). This agrees with the report of Ouattara *et al.* [31] *,* but disagrees with that of Aderaw and Gedefaw [19] who reported a very low rate of ITN awareness as a method of malaria control in Amahara National Regional State, Ethiopia*.* However, only 1% of respondents knew about the insecticidal properties of ITNs. This could be a draw back in the practice of treating/re-treating and even the utilization of ITNs. Five (5)% of respondents used ITNs mainly for warmth, which suggests that in the dry season, utilization may drop because of increased heat. Sensitization messages need to be intensified for a change of behaviour to use ITNs at all times. The utilization of ITNs by the vulnerable populations was however not associated with the knowledge of malaria and awareness of ITNs as a preventive measure against malaria.

The study had as limitation recruiting the appropriate sample sizes for some of the communities as the population size given by the Buea health service did not reflect the actual populations on the field. However, this had no significant effect on the findings when the samples were pooled together.

## Conclusions

In conclusion, malaria was generally perceived as a dangerous disease with life threatening effects on children under five years and pregnant women. The level of knowledge on the transmission of malaria and the level of awareness of ITNs as a preventive measure against malaria was high among the household members in the BHD. Even though the level of knowledge of malaria and the awareness of ITNs was quite high among pregnant women and mothers/caretakers of under-fives, some individuals still had wrong beliefs about the cause and prevention of malaria. There is therefore a need to develop new strategies for sensitization messages to reach every community member especially those with no formal education. Such interventions should involve the active participation of members of the community and should lay emphasis on different kinds of malaria control methods such as personal protection, draining of stagnant water around homes and the use of insecticide residual spraying.

## Authors’ information

HKK: PhD and Associate Professor of Medical Parasitology, Head of Department, Zoology and Animal Physiology. SBN: MPh in public health. JLN-N: MSc and Assistant Lecturer of Zoology. IUNS: PhD and Lecturer of Parasitology. JA: PhD and Lecturer of Public Health. MBSA: PhD and Lecturer of Nursing, Head of Department, Nursing.

## Abbreviations

BHD: Buea Health District
CRAs: Community relay agents
ITNs: Insecticide-treated bed nets
OR: Odds ratio.
